# Pancreatic Tumor Organoid-Derived Factors from Cachectic Patients Disrupt Contractile Smooth Muscle Cells

**DOI:** 10.3390/cancers16030542

**Published:** 2024-01-26

**Authors:** Rianne D. W. Vaes, Annemarie A. van Bijnen, Steven W. M. Olde Damink, Sander S. Rensen

**Affiliations:** 1 Department of Surgery, NUTRIM School of Nutrition and Translational Research in Metabolism, Maastricht University, 6229 ER Maastricht, The Netherlands; 2 Department of General, Visceral and Transplant Surgery, RWTH Aachen University Hospital, 52074 Aachen, Germany

**Keywords:** sarcopenia, cachexia, phenotypic modulation, synthetic smooth muscle cell phenotype, organoids

## Abstract

**Simple Summary:**

Pancreatic cancer is associated with weight loss and negatively affects skeletal muscle function. It is not known whether the changes observed in skeletal muscle also occur in smooth muscle, a tissue critical for gastrointestinal functioning. In cancer-related muscle wasting, it is widely assumed that both tumor- and host-derived factors and pro-inflammatory cytokines directly target skeletal muscle. This study aimed to investigate the impact of pancreatic tumor-derived factors on smooth muscle. The results from this study show that pancreatic tumor organoid-derived factors compromise the contractile phenotype of healthy smooth muscle cells. Loss of the contractile smooth muscle cell phenotype may impair the contractile functionality of the intestinal smooth musculature and may therefore contribute to the frequently reported gastrointestinal symptoms in pancreatic cancer patients.

**Abstract:**

Patients with pancreatic cancer often suffer from cachexia and experience gastrointestinal symptoms that may be related to intestinal smooth muscle cell (SMC) dysfunction. We hypothesized that pancreatic tumor organoids from cachectic patients release factors that perturb the SMC’s contractile characteristics. Human visceral SMCs were exposed to conditioned medium (CM) from the pancreatic tumor organoid cultures of cachectic (*n* = 2) and non-cachectic (*n* = 2) patients. Contractile proteins and markers of inflammation, muscle atrophy, and proliferation were evaluated by qPCR and Western blot. SMC proliferation and migration were monitored by live cell imaging. The Ki-67-positive cell fraction was determined in the intestinal smooth musculature of pancreatic cancer patients. CM from the pancreatic tumor organoids of cachectic patients did not affect *IL-1β*, *IL-6*, *IL-8*, *MCP-1*, or *Atrogin-1* expression. However, CM reduced the α-SMA, γ-SMA, and SM22-α levels, which was accompanied by a reduced SMC doubling time and increased expression of *S100A4*, a Ca^2^+-binding protein associated with the synthetic SMC phenotype. In line with this, Ki-67-positive nuclei were increased in the intestinal smooth musculature of patients with a low versus high L3-SMI. In conclusion, patient-derived pancreatic tumor organoids release factors that compromise the contractile SMC phenotype and increase SMC proliferation. This may contribute to the frequently observed gastrointestinal motility problems in these patients.

## 1. Introduction

Cachexia is a multifactorial wasting syndrome that is present in up to 80% of pancreatic cancer patients [1]. It is defined as a complex metabolic syndrome that is characterized by body weight loss predominantly due to the progressive loss of muscle mass, which may or may not be accompanied by the loss of adipose tissue. The consensus definition of cancer cachexia is based on having >5% body weight loss over the last six months, or having >2% body weight loss in combination with a BMI < 20 or sarcopenia [2]. Systemic inflammation and hypermetabolism play important roles in the pathogenesis of cancer cachexia. Cachexia is associated with reduced physical function, reduced tolerance to chemo- and radiotherapy, and reduced quality of life [2]. Moreover, it greatly contributes to the poor prognosis of pancreatic cancer patients [1,3].

The involuntary loss of body mass in cancer cachexia mainly concerns skeletal muscle, which accounts for 40% of the total body weight. In response to systemic perturbations induced by the tumor (e.g., catabolic stimuli, elevated pro-inflammatory cytokines), muscle atrophy occurs as the result of a disturbed balance between anabolic and catabolic processes. Catabolic stimuli induce muscle-specific E3 ubiquitin ligases MuRF1 and Atrogin-1/MAFbx to bind to selective substrates, thereby targeting them for ubiquitination and subsequent degradation by the 26S proteasome [4]. Similar alterations have recently been observed in the cardiac muscle of cachectic patients, suggesting that these pathways are also activated in cardiac muscle to induce muscle wasting [5,6].

At present, it is not known whether the changes found in atrophying skeletal and cardiac muscle in response to tumor-derived factors also occur in smooth muscle, the third muscle compartment, which fulfils a crucial role in human physiology. However, it is known that smooth muscle cells (SMCs) may also respond to atrophy-inducing stimuli by increasing ubiquitin ligase expression [7,8]. For example, Bdolah et al. showed that both MuRF1 and Atrogin-1/MAFbx are upregulated in the involuting uterine smooth muscle following the delivery of the fetus, raising the possibility that activation of MuRF1 and Atrogin-1/MAFbx may be a common phenomenon in cells undergoing atrophy [7]. Moreover, it has previously been shown that in response to inflammatory stimuli, SMCs can produce and release pro-inflammatory cytokines [9,10]. As systemic inflammation is suggested to play a key role in cancer cachexia-induced muscle wasting, SMCs may be targeted by these circulating inflammatory cytokines and play a role in sustaining it as well.

In contrast to skeletal and cardiac muscle cells that differentiate terminally, mature contractile SMCs retain remarkable plasticity, as expressed by their ability to undergo so-called phenotypic modulation in response to injury and disease. Typical contractile SMCs are characterized by an elongated spindle-shaped morphology and the high expression of a unique repertoire of contractile proteins. Loss of this contractile phenotype is accompanied by morphological alterations resulting in round-shaped SMCs, referred to as epithelioid or rhomboid, as well as increased proliferation rates, high migration rates, and the increased synthesis of extracellular matrix (ECM) proteins [11].

Contractile SMCs are the force-producing cells that are essential for gut motor activity, required to move nutrients through the gastrointestinal (GI) tract [11]. Diminished contractile SMC function results in profound GI motility problems [12] that may contribute to nutritional-related complications in cancer cachexia. Indeed, Zhou and colleagues recently identified symptoms of early satiety, diarrhea, and constipation in cachectic cancer patients that were suggested to be secondary to gastrointestinal (GI) smooth muscle dysfunction [13]. In further support of a role for smooth muscle in the pathophysiology of cachexia, we recently showed several aberrations in the intestinal smooth muscle of pancreatic cancer patients with sarcopenia, including the reduced expression of smoothelin, a key smooth muscle contractile protein [14]. Moreover, pronounced collagen deposition around the myenteric plexus was observed in these patients, in line with the increased ECM protein expression by synthetic SMCs. This may further disturb the generation of the contractile forces that are regulated by the enteric nervous system.

In view of these recent data, we hypothesized that the factors released by the pancreatic tumor cells of cachectic patients induce the phenotypic modulation of healthy contractile SMCs to their synthetic counterparts. Recent advances in the modeling of pancreatic cancer using three-dimensional (3D) organoid cultures enable a novel approach to studying this hypothesis [15,16]. Pancreatic tumor organoids self-organize into structures closely mimicking the tumor and accurately recapitulate many relevant aspects of disease progression in vitro and in vivo [15,17]. Importantly, the cachexia severity of patients can be thoroughly assessed before establishing their organoid cultures, which is crucial to identify and better understand cachexia-inducing mechanisms in humans. In the current study, we aimed to show that key characteristics of contractile visceral SMCs are adversely impacted by pancreatic tumor cell factors. We used our recently established pancreatic tumor organoid biobank [18] to obtain tumor-derived factors from both cachectic and non-cachectic patients. Our validated human visceral SMC culture system [19] was subsequently used to assess the effect of these tumor organoid-derived factors on the contractile SMC phenotype, and key findings were validated using intestinal smooth muscle tissue from pancreatic cancer patients with and without sarcopenia.

## 2. Materials and Methods

### 2.1. Human Pancreatic Tumor Organoid Culture

Patient-derived pancreatic tumor organoid cultures PANCO-9a, PANCO-11a, PANCO-12a, and PANCO-17a were previously generated and characterized in our laboratory [18]. All patients were diagnosed with pancreatic ductal adenocarcinoma (PDAC), for which they underwent a Whipple procedure (also known as pancreaticoduodenectomy), a major surgical procedure involving the removal of the head of the pancreas, where the tumor is located. Ethical approval (METC 13-4-107) was obtained to pre-operatively assess the cachexia status of the pancreatic cancer patients and to obtain a tumor biopsy from the resection material. In short, surgically removed pancreatic tumor tissue was immediately transferred to the pathology laboratory (Department of Pathology, MUMC+), where a dedicated gastrointestinal pathologist identified the tumor macroscopically and collected a fresh, approximately 0.5–1 cm^3^ tumor-containing tissue slice. The tumor tissue slice was minced, washed with ice-cold Advanced Dulbecco’s Modified Eagle medium/Ham’s F-12 (AdvDF+++) (Gibco, Paisley, UK, Cat. No. 12634-010) supplemented with 1× GlutaMax (Gibco, Cat. No. 35050-061), 10 mM HEPES (Gibco, Cat. No. 15630-080), and Pen/Strep (50 units/mL penicillin and 50 μg/mL streptomycin) (Gibco, Cat. No. 15140-122), and digested with collagenase II (5 mg/mL, Gibco, Cat. No. 17101-01) on an orbital shaker at 37 °C for 1–2 h. The digested tissue suspension was further digested with TrypLE (Gibco, Cat. No. 12605-010). The resulting cell suspension was centrifuged for 5 min at 350× *g* at 4 °C and the cell pellet was resuspended in ice-cold BME (Geltrex LDEV-free reduced growth factor basement membrane matrix, Gibco, Cat. No. 1413202). For the maintenance of cultures, organoids were resuspended in ice-cold basement membrane extract (BME; Geltrex LDEV-Free Reduced Growth Factor Basement Membrane Matrix, Gibco, Cat. No. 14132) and three ~15 μL droplets of the BME–cell suspension were allowed to solidify per well of a 24-well culture plate (Eppendorf, Hamburg, Germany) at 37 °C for 30 min. When the droplets were solidified, 500 μL of organoid medium was added to each well [18]. The plate was transferred to a humidified 37 °C/5% CO_2_ incubator and the medium was changed every 2–3 days. The organoids were passaged every 7–10 days.

### 2.2. Collection of Pancreatic Tumor Organoid Conditioned Medium

One day before passaging, the organoid growth medium was replaced by a basic culture medium consisting of DMEM/F12 supplemented with 1% (*v*/*v*) HEPES and 1% (*v*/*v*) Pen/Strep. Additional wells containing empty BME droplets overlaid with DMEM/F12 were included for the collection of the control medium. The medium was conditioned for 24 h. After 24 h, the conditioned medium (CM) was collected and centrifuged at 350× *g* for 10 min at 4 °C. The supernatant was centrifuged for another 20 min at 2000× *g* at 4 °C and the resulting CM cleared of cellular debris was aliquoted and stored at −80 °C. The CM used for experiments was collected between passage numbers 6 and 15.

### 2.3. Smooth Muscle Cell Culture

Human uterine SMCs (ULTR cells) [20] were cultured in Dulbecco’s Modified Eagle’s Medium (DMEM) (Gibco, Cat. No. 42430) supplemented with 10% (*v*/*v*) fetal bovine serum (FBS) (Greiner Bio-One, Cat. No. 758093), 4 mM L-Glutamine, and antibiotics (100 units/mL penicillin and 100 μg/mL streptomycin, Gibco). The cells were maintained at 37 °C, 5% CO_2_ in a humidified incubator.

For experiments, SMCs were cultured according to our previously published method that generates SMCs with a highly contractile phenotype. These contractile SMCs have an elongated spindle-shaped morphology and increased expression levels of SMC-specific contractile proteins, including SM-MHC, α-SMA, and SM22α [19]. In short, SMCs were plated at a density of 1 × 10^4^ cells/cm^2^ on BME-coated surfaces. When cells reached >90% confluency after 48 h, the regular culture medium was replaced by advanced DMEM/F-12 supplemented with 2% (*v*/*v*) FBS and antibiotics (50 units/mL penicillin and 50 μg/mL streptomycin, Gibco). The medium was refreshed every three days. At day 6, SMCs were treated with 50% (*v*/*v*) pancreatic tumor organoid-derived CM.

### 2.4. Quantitative Real-Time PCR

Total RNA was isolated using TRI Reagent (Sigma, St. Louis, MO, USA) according to the manufacturer’s instructions. The RNA yield was measured with a DeNovix DS-11 spectrophotometer and 375 ng RNA was reversed-transcribed to cDNA using the SensiFast cDNA Synthesis Kit, according to the manufacturer’s protocol (Bioline GmbH, London, UK). The gene expression levels of SMC phenotype markers were quantified using a three-step PCR program followed by melting curve analysis using the LightCycler480 (Roche, Mannheim, Germany). cDNA was amplified with the SensiMix SYBR Hi-Rox kit (Bioline GmbH, Cat. No. QT605-05). Specific primer pairs for each gene were ordered from Sigma and are presented in Table S1. Relative gene expression levels were derived from the LinRegPCR (version 2016.1) method [21]. Normalization was performed by dividing the relative gene expression levels of each target by the geometric mean of two reference genes, cyclophylin A (CYPA) and beta-2-microglobulin (B2M).

### 2.5. Western Blotting

After contractile SMCs (day 6) were exposed to 50% (*v*/*v*) pancreatic tumor organoid-derived CM for 72 h, cells were harvested in lysis buffer containing 10 mM Tris, 100 mM NaCl, 1 mM EDTA, 1 mM EGTA, 1% Triton X-100, 10% glycerol, 0.1% (*w*/*v*) SDS, and 0.5% (*v*/*v*) sodium deoxycholate supplemented with protease inhibitor cocktail tablets (Roche). The Western blot analysis was performed as described previously [19]. In short, PVDF membranes were incubated overnight at 4 °C with specific monoclonal primary antibodies directed against anti-smooth muscle myosin heavy chain (SM-MHC) (1:1000, Clone BT-562, Alfa Aesar, Cat. No. J64817AMJ), anti-α-smooth muscle actin (α-SMA) (1:1000, Clone 1A4, Dako, Santa Clara, CA, USA, Cat. No. M0851), or anti-smooth muscle protein 22-alpha (SM22α) (1:1000) (clone 3E11, kind gift from Dr. A. Chiavegato, University of Padua, Italy). After washing with TBS-Tween20 (0.01%), the blots were probed for 1 h with an appropriate peroxidase-conjugated secondary antibody (Vector Laboratories, Newark, CA, USA) and signals were visualized using the SuperSignal West Pico chemiluminescent substrate (Thermo Scientific, Rockford, IL, USA) according to the manufacturer’s instructions. Images were obtained with a molecular imager (Amersham Imager 600, GE Healthcare Life Sciences, Chicago, IL, USA) and the total band intensity was quantified with the ImageQuant TL software (v8.1.0.0, GE Healthcare Life Sciences). Normalization was performed by dividing the total band intensity of the protein of interest by the total band intensity of tubulin. The original western blot figures can be found in File S1.

### 2.6. IncuCyte™ Cell Confluence Proliferation Assay

SMCs were plated in 96-well cell culture plates (Eppendorf) at a density of 1 × 10^4^ cells/cm^2^ on BME-coated surfaces. After 3 h, the cells were attached to the surface and the growth medium was replaced by 50% (*v*/*v*) pancreatic tumor organoid-derived CM. Cell confluency was monitored by the IncuCyte^®^ S3 Live-Cell Analysis System (Sartorius, Göttingen, Germany). Phase-contrast images were captured every 2 h using the 10× objective. Quantification of the occupied area (% confluency) of all individual images over time was performed by using the integrated cell confluence analysis tool. The doubling time was calculated based on the linear part of the proliferation curve.

### 2.7. IncuCyte™ Scratch Wound Cell Migration Assay

For the migration assay, SMCs were plated at a density of 1 × 10^4^ cells/cm^2^ on BME-coated IncuCyte^®^ ImageLock 96-well microplates. Subsequently, the SMCs were cultured as previously described [19]. At day 6, homogeneous scratch wounds (700–800 μm) were introduced by using the IncuCyte^®^ WoundMaker tool (Sartorius, Göttingen, Germany), according to the manufacturer’s instructions. A cell-free zone was created within the confluent monolayer of SMCs in each well. Cell confluency within the cell-free zone was monitored by the IncuCyte^®^ FLR system (2011A Rev2, Essen Bioscience, Ann Arbor, MI, USA). Phase-contrast images were captured every 2 h using the 10× objective. The Relative Wound Density v1.0 algorithm that was integrated into the software (2011A Rev2, Essen Bioscience) was used to quantify the spatial cell density in the wound area relative to the spatial cell density outside of the wound area at every time point. This metric is self-normalizing for changes in cell density that may occur outside the wound as a result of cell proliferation and does not rely on the identification of cell boundaries.

### 2.8. Patient Cohort

Archived formalin-fixed paraffin-embedded (FFPE) jejunum tissue sections from twenty-two pancreatic cancer patients who underwent a Whipple procedure between 2009 and 2013 were obtained from the Pathology Department at the Maastricht University Medical Center (MUMC+), The Netherlands. Since the degree of weight loss was not always available in the medical records of these patients, and because self-reported weight loss data are frequently unreliable [22], we classified patients as cachectic or not cachectic based on the presence of sarcopenia, a hallmark of cachexia. Sarcopenia was quantitatively assessed by analyzing the L3 skeletal muscle index (L3-SMI) using pre-operative computed tomography (CT) scans at the level of the L3 vertebra, as previously described [23]. The L3-SMI has been shown to be a good measure of total body skeletal muscle mass [24]. Sex-specific median cut-off values for L3-SMI were used to assign patients to either a high L3-SMI (sarcopenic/cachectic) or low L3-SMI (non-sarcopenic/non-cachectic) group (Table S2).

### 2.9. Immunohistochemical Staining and Evaluation

Paraffin-embedded jejunum tissue sections (4 μm) were deparaffinized through xylene, treated with 0.6% (*v*/*v*) H_2_O_2_ for 15 min to block endogenous peroxidase activity, and rehydrated through graded ethanol to water. Subsequently, the tissue sections were incubated for 20 min (90–95 °C) with target retrieval solution according to the manufacturer’s instructions (DAKO, #S1699). The sections were rinsed with PBS and blocked for 30 min with 5% BSA in 1xPBS to reduce background staining. Tissue sections were incubated with a monoclonal mouse anti-human Ki-67 antibody (1:200 dilution) (Clone MIB-1, Dako #M7240). Subsequently, the tissue sections were washed and incubated with a rabbit anti-mouse IgG biotin-labelled secondary antibody (Dako, Cat. No. 0413). The Vectastain Reagent (VectaStain Elite ABC-HRP kit, Vector Laboratories, Cat. No. PK-6200) was used for detection. Peroxidase substrate solution (3,3′-Diaminobenzidine, DAB) (Dako) was used to visualize the presence of the peroxidase enzyme. Sections were counterstained for 1 min with hematoxylin (Merck, Darmstadt, Germany), washed, and then mounted with Entellan (Merck).

Stained jejunal tissue slides were digitalized using the Ventana iScan HT (Version 1.1, Roche, Ventana Medical Systems, Inc., Oro Valley, AZ, USA) using a 200× magnification. The scans were opened in the Pannoramic Viewer software (version 1.15.4, 3DHISTECH, Ltd., Budapest, Hungary) for the selection of regions of interest (ROI). At least 5 representative ROIs were selected in the transversal section of the circular smooth muscle layer (CL).

Selected ROIs were converted to TIFF files and imported in QuPath (v0.1.2) [25]. Positive and negative stained nuclei were counted and the percentage of Ki-67-positive stained nuclei was calculated for each individual ROI.

### 2.10. Statistical Analysis

All data were obtained from three independent experiments performed in triplicate and were analyzed using IBM SPSS 25 for Microsoft Windows^®^ (IBM Corp. Released 2017. IBM SPSS Statistics for Windows, Version 25.0. Armonk, NY, USA: IBM Corp). Results are expressed as mean ± SEM and statistical analyses were performed using the independent-sample *t*-test to compare differences between two groups. In case of more than two groups, the one-way ANOVA test was used, followed by Tukey’s post-hoc testing. Fisher’s exact test was used for between-group comparisons of categorical variables. The migration data were analyzed with a two-way ANOVA repeated-measures test followed by Dunnett’s multiple-comparison test. A *p*-value of *p* < 0.05 was considered statistically significant.

## 3. Results

### 3.1. Tumor Organoid Factors from Non-Cachectic Pancreatic Cancer Patients Increase Pro-Inflammatory Cytokines in Human Visceral SMCs

**Figure 1:**
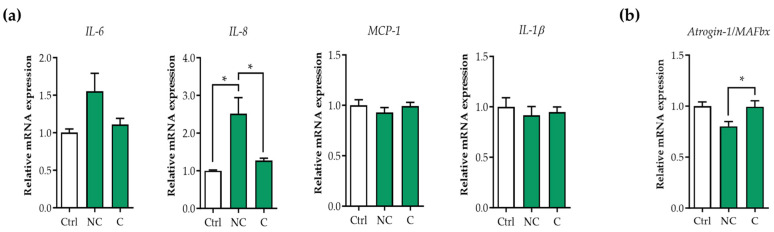
Tumor organoid factors from non-cachectic pancreatic cancer patients induce upregulation of pro-inflammatory cytokines by human SMCs. SMCs were cultured for 6 days on BME-coated surfaces and with 2% FBS to induce a contractile phenotype. SMCs were subsequently treated with 50% (*v*/*v*) control medium (DMEM/F12) or 50% (*v*/*v*) pancreatic tumor organoid-derived CM from cachectic (C) and non-cachectic (NC). (**a**) mRNA expression levels of pro-inflammatory cytokines (*IL-6, IL-8, MCP-1*, and *IL-1β*) and (**b**) E3-ubiquitin ligase *Atrogin-1/MuRF1* were determined after 48h. Data were normalized to *CYPA* and *B2M* reference genes. Relative expression levels were obtained from three independent experiments. Data are presented as mean ± SEM. A *p*-value less than 0.05 was considered to be statistically significant and is indicated through an asterisk (*). *IL-6*, interleukin-6; *IL-8*, interleukin-8; *MCP-1*, monocyte chemoattractant protein-1; *IL-1β*, interleukin-1β.

To investigate the effect of tumor-derived factors from pancreatic cancer patients on SMCs, we selected four recently generated pancreatic tumor organoid cultures from cachectic (PANCO-9a, PANCO-17a) and non-cachectic patients (PANCO-11a, PANCO-12a) [18]. We first assessed whether tumor organoid factors from cachectic patients induced the expression of pro-inflammatory cytokines in SMCs (Figure 1a). Whereas the tumor CM of non-cachectic patients induced the expression of interleukin-8 (*IL-8*) (2.5-fold, *p* = 0.010), this was not observed with the CM of the cachectic patients. Similarly, *IL-6* levels tended to be increased by the tumor secretome of non-cachectic patients (1.6-fold, *p* = 0.14) but not by that of the cachectic patients. *Mcp-1* and *IL-1β* expression was not affected by tumor factors from either cachectic or non-cachectic patients.

### 3.2. E3 Ubiquitin Ligases Are Not Upregulated in Human Visceral SMCs Exposed to Pancreatic Tumor Organoid-Derived CM

To investigate whether tumor organoid-derived factors induced the activation of the ubiquitin proteasome system in SMCs, as they do in skeletal muscle, we exposed contractile SMCs to the pancreatic tumor organoid CM for 48 h. Whereas *MuRF1* expression could not be detected, *Atrogin-1/MAFbx* was detectable but not increased compared to the control condition (Figure 1b). This suggests that the ubiquitin proteasome pathway is not activated in response to pancreatic tumor organoid-derived factors from either cachectic or non-cachectic patients.

### 3.3. Tumor Organoid Factors from Cachectic Pancreatic Cancer Patients Diminish Contractile Protein Levels in SMCs

**Figure 2:**
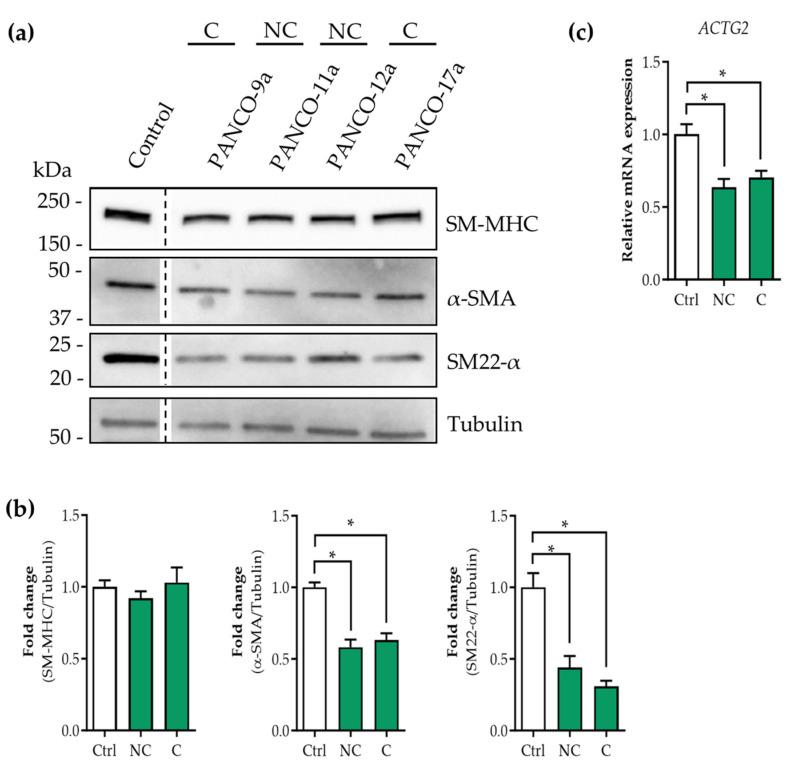
Tumor organoid factors from cachectic pancreatic cancer patients decrease SMC contractile proteins. Human SMCs were cultured for 6 days on BME-coated surfaces and with 2% FBS to induce high expression of contractile SMC markers. At day 6, SMCs were treated with 50% (*v*/*v*) pancreatic tumor organoid-derived CM from cachectic (C: PANCO-9a and PANCO-17a) and non-cachectic (NC: PANCO-11a and PANCO-12a) patients. (**a**) Protein levels of smooth muscle myosin heavy chain (SM-MHC), α-smooth muscle actin (α-SMA), and smooth muscle protein 22-α (SM22α) were determined by Western blot after 72 h. (**b**) Protein levels were normalized to α-tubulin and control was set at 1. (**c**) mRNA expression of γ-smooth muscle actin (ACTG2) was determined after 48 h. Data were normalized to CYPA and B2M reference genes. Protein and relative gene expression levels were obtained from three independent experiments. Data are presented as mean ± SEM. A *p*-value less than 0.05 was considered to be statistically significant and is indicated through an asterisk (*).

To investigate whether SMCs undergo phenotypic modulation in response to factors derived from the pancreatic tumor cells of cachectic pancreatic cancer patients, contractile SMCs were exposed to tumor organoid CM for 72 h and the levels of key contractile proteins (smooth muscle myosin heavy chain (SM-MHC), α-smooth muscle actin (α-SMA), and smooth muscle protein 22-alpha (SM-22α)) were assessed by Western blot. Although no differences in SM-MHC protein levels were observed, both α-SMA (no-cachexia (NC): −1.7-fold, *p* < 0.001; cachexia (C): −1.6-fold, *p* < 0.001) and SM22-α (NC: −2.3-fold, *p* < 0.001; C: −3.2-fold, *p* < 0.001) protein levels were markedly decreased by the tumor organoid secretome of both cachectic and non-cachectic patients compared to the control condition (Figure 2a,b). In line with this, the mRNA expression of γ-smooth muscle actin (*ACTG2*), the dominant actin isoform in visceral SMCs [26], was also significantly reduced compared to the control (NC: −1.6-fold, *p* < 0.001; C: −1.4-fold, *p* < 0.001) (Figure 2c). These results indicate that tumor-derived factors negatively affect the contractility of SMCs, consistent with phenotypic modulation to the synthetic phenotype.

### 3.4. Tumor Organoid-Derived Factors Induce Proliferation but Not Migration of Contractile Human Visceral SMCs

**Figure 3:**
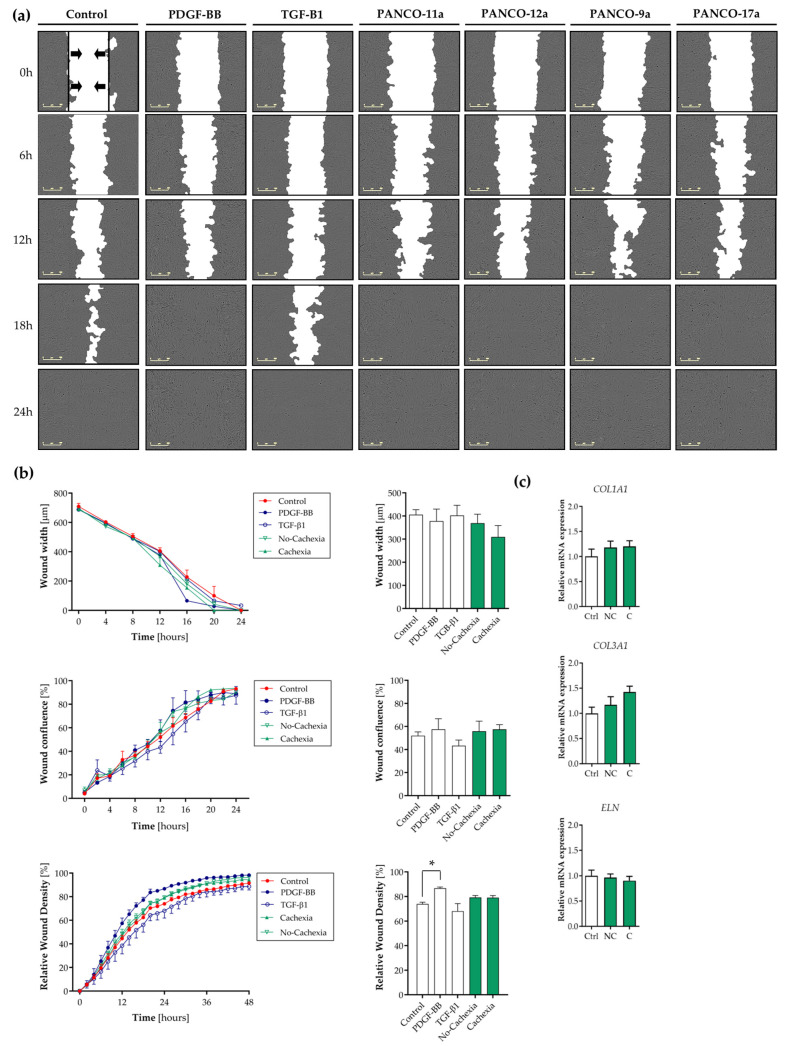
Tumor organoid-derived factors promote SMC proliferation but do not affect SMC migration. SMCs were cultured for 6 days on BME-coated surfaces and with 2% FBS to induce a contractile phenotype. At day 6, homogeneous scratch wounds were introduced. Immediately thereafter, contractile SMCs were treated with 50% (*v*/*v*) pancreatic tumor organoid-derived CM from cachectic (C: PANCO-9a and PANCO-17a) and non-cachectic (NC: PANCO-11a and PANCO-12a) patients for 48 h. Wound closure was measured in real time over a period of 48 h. (**a**) Representative phase-contrast images. Scale bar = 300 μm. Black arrows indicate the cell-free zone. (**b**) Migration of human visceral SMCs is plotted as relative wound density (%) vs. time (hours). Data are presented as mean ± SEM; *n* = 3 for each time point. Data were obtained from three independent experiments. (**c**) mRNA expression of genes encoding the ECM proteins collagen I (*COL1A1*), collagen III (*COL3A1*), and elastin (*ELN*) were determined after 48 h. Data were normalized to *CYPA* and *B2M* reference genes. Relative gene expression levels were obtained from three independent experiments. Data are presented as mean ± SEM. A *p*-value less than 0.05 was considered to be statistically significant and is indicated through an asterisk (*).

To confirm that tumor organoid-derived factors caused a shift towards the synthetic SMC phenotype, we next assessed the key characteristics of synthetic SMCs, including their migration and proliferation rates and the synthesis of ECM proteins. First, we performed a scratch wound assay on a monolayer of contractile SMCs in the presence or absence of tumor organoid CM. PDGF-BB (a well-known inducer of SMC proliferation and migration) and TGF-β1 (a known inducer of the contractile SMC phenotype) were included as controls. The real-time monitoring of SMC migration revealed that the cell-free zone was covered with cells within 24 h of wounding in all conditions (Figure 3a). As expected, the time needed for wound closure was reduced in SMCs treated with PDGF-BB compared to the control condition (Figure 3a,b, *p* = 0.005). The opposite effects were observed with TGF-β1. The tumor organoid CM of both cachectic and non-cachectic patients increased SMC migration, as indicated by the relative wound density plot (Figure 3b), although the difference from the control medium was not statistically significant (*p* = 0.41 and *p* = 0.21 versus control, respectively).

To investigate whether the SMCs increased the levels of ECM proteins in response to pancreatic tumor organoid factors, the expression of genes encoding three major ECM proteins (collagen I (*COL1A1*), collagen III (*COL3A1*), and elastin (*ELN*)) was measured. Although *COL3A1* expression tended to be increased after exposure to the tumor organoid CM of cachectic patients compared to the control condition (1.4-fold, *p* = 0.16), no significant differences in SMC *COL1A1*, *COL3A1*, or *ELN* mRNA expression were observed in SMCs exposed to tumor factors from cachectic or non-cachectic patients (Figure 3c).

**Figure 4:**
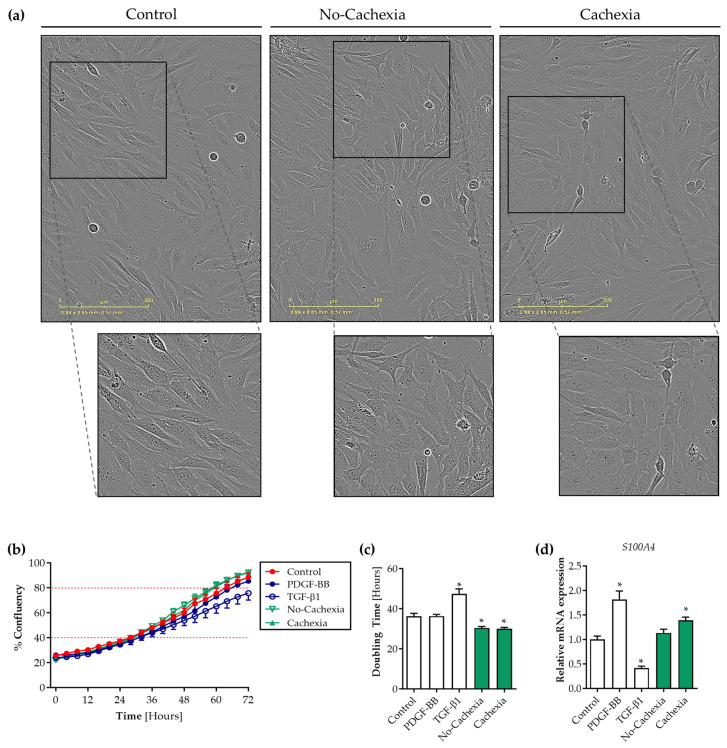
Tumor organoid-derived factors induce proliferation of contractile human SMCs. SMCs were treated with either control medium, PDGF-BB, TGF-β1, or 50% (*v*/*v*) pancreatic tumor organoid-derived CM from cachectic (PANCO-9a, PANCO-17a) and non-cachectic (PANCO-11a, PANCO-12a) patients. Cell confluency was monitored and phase-contrast images were captured every 2 h. (**a**) Representative phase-contrast images after 72 h, showing rhomboid morphology of SMCs exposed to tumor factors from cachectic patients. Scale bar = 200 μm. (**b**) Proliferation of human visceral SMCs plotted as percentage (%) confluency vs. time (hours). (**c**) The doubling time was calculated for each individual group. The doubling time indicates the time that SMCs require to double in number in the exponential growth phase (40–80% confluency). (**d**) mRNA expression of S100A4 was determined in SMCs cultured for 6 days on BME-coated surfaces and with 2% FBS to induce contractile marker expression. These SMCs were treated for 48 h with control medium or 50% (*v*/*v*) pancreatic tumor organoid-derived CM. Data were normalized to CYPA and B2M reference genes. Data were obtained from three independent experiments. Data are presented as mean ± SEM. A *p*-value less than 0.05 was considered to be statistically significant and is indicated through an asterisk (*).

Next, we assessed the proliferation rate of SMCs in response to tumor organoid-derived factors. Cells were seeded at ~20% confluency and exposed to the tumor organoid CM or control medium, and confluency as a measure of proliferation was assessed over the next 72 h. Interestingly, while monitoring the SMCs by live cell imaging, pronounced differences in their morphology were observed when cultured in the presence of the tumor organoid CM (Figure 4a). Whereas SMCs in the control medium predominantly adopted elongated spindle shapes reminiscent of a contractile phenotype, SMCs exposed to the pancreatic tumor organoid-derived factors of cachectic and non-cachectic patients were predominantly rhomboid-shaped. Moreover, we observed that the tumor organoid CM markedly accelerated the coverage of the culture area by SMCs (Figure 4b and Videos S1–S5). This observation was confirmed by the significantly reduced time that contractile SMCs required to double in number in the exponential growth phase (40–80% confluency) in the presence of the tumor organoid CM from both cachectic and non-cachectic patients compared to the control (control: 36.2 h vs. no-cachexia 30.4 h, *p* = 0.007 and vs. cachexia: 30.0 h, *p* = 0.003) (Figure 4c). As expected, TGF-β1 significantly increased the doubling time (control: 36.2 h vs. TGF-β1: 47.4 h, *p* < 0.001). To provide further support for the proliferative effect of tumor organoid factors on SMCs, we also assessed the expression of *S100A4*, a Ca^2+^-binding protein associated with SMC proliferation [27,28]. In line with the live cell imaging data, *S100A4* mRNA expression was significantly increased by the exposure of contractile SMCs to pancreatic tumor organoid factors from cachectic patients (1.4-fold, *p* = 0.02) (Figure 4d). Together, these data show that pancreatic tumor organoid factors promote SMC proliferation, whereas the migration and synthesis of ECM proteins are not affected.

### 3.5. Sarcopenia in Pancreatic Cancer Patients Is Associated with Increased Intestinal Smooth Muscle Proliferation

**Figure 5:**
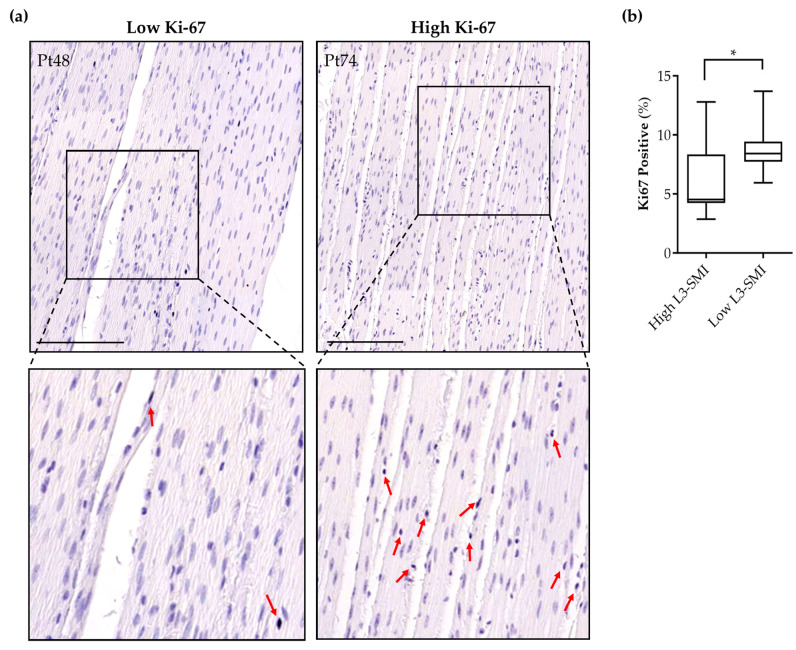
Increased intestinal smooth muscle proliferation in pancreatic cancer patients is associated with sarcopenia. Retrospective analysis of SMC proliferation in the intestinal smooth musculature of twenty-two pancreatic cancer patients. (**a**) Representative images of Ki-67-stained nuclei in the smooth musculature of patients with a high L3-SMI (left panel) or a low L3-SMI (right panel). Red arrows indicate Ki-67-positive nuclei. Hematoxylin (blue/purple) was used as a nuclear stain. Scale bar = 200 μm. (**b**) The percentage of Ki-67-positive nuclei is represented in a box plot graph. The boxes represent the interquartile range (IQR), in which the medians are indicated as a bar. Differences between patients with a high L3-SMI (*n* = 11) and a low L3-SMI (*n* = 11) were analyzed using the non-parametric Mann–Whitney U test, * *p* < 0.05.

To examine whether the induction of the proliferative SMC phenotype by tumor-derived factors observed in vitro could be validated in human tissue, we analyzed the Ki-67-positive cell numbers in the intestinal smooth musculature of twenty-two pancreatic cancer patients. Patient characteristics are presented in Table 1. Based on the median sex-specific L3-SMI cut-off values, patients were assigned to a high L3-SMI (*n* = 11) or a low L3-SMI (*n* = 11) group. A significantly higher percentage of Ki-67-positive nuclei was found in the intestinal smooth musculature of pancreatic cancer patients with a low L3-SMI compared to those patients with a high L3-SMI (8.7 ± 2.1% vs. 6.4 ± 3.2% (*p* = 0.047), respectively) (Figure 5a,b). These data imply a shift towards a proliferative, synthetic SMC phenotype in the intestinal musculature of pancreatic cancer patients with a low L3-SMI, which is a key characteristic of cancer cachexia.

## 4. Discussion

Cancer-induced muscle wasting is known to involve both striated muscle compartments, i.e., skeletal muscle and cardiac muscle [5,6]. At present, it is not known whether the aberrations found in atrophying skeletal and cardiac muscle in response to tumor-derived stimuli also occur in smooth muscle. Here, we showed that tumor organoid-derived factors from both cachectic and non-cachectic pancreatic cancer patients strongly reduced the expression of proteins involved in SMC contraction, reflecting the loss of the SMC contractile phenotype. In parallel, the proliferation of human visceral SMCs was accelerated, accompanied by the increased expression of S100A4 only after stimulation with tumor factors from cachectic patients. In line with these in vitro data, we detected an increased number of proliferating SMCs in the intestinal smooth musculature of pancreatic cancer patients with a low skeletal muscle mass. Overall, these data show that human contractile SMCs undergo phenotypic modulation in response to tumor-derived factors from pancreatic cancer patients, translating into diminished contractile potential.

Muscle wasting in cancer cachexia is considered to be induced by tumor-derived factors that directly or indirectly activate intracellular signaling pathways, disturbing cell homeostasis. In contrast to skeletal muscle, which is targeted for degradation by the muscle-specific E3 ubiquitin ligases MuRF1 and Atrogin1/MAFbx [4], no upregulation of either of these E3 ligases was detected in human visceral SMCs treated with tumor organoid factors from cachectic pancreatic cancer patients. This is in line with our recent data showing that tumor organoid factors from pancreatic cancer patients did not activate skeletal muscle atrophy signaling pathways in mature C2C12 myotubes [29]. A distinguishing feature of mature contractile SMCs is that they retain the capacity to reversibly modulate their phenotype in response to a variety of stimuli [30] and, therefore, SMCs may respond differently to tumor-derived factors compared to skeletal muscle cells. In line with this notion, we showed that mature visceral SMCs expressed lower levels of contractile SMC-specific proteins after exposure to tumor organoid-derived factors, indicating that they lose their contractile phenotype. The loss of such SMC-specific proteins that are part of the contractile machinery of visceral SMCs can result in severe contractile impairment and consequent GI dysmotility [11,31]. In cancer patients, this may have potentially serious consequences, including malnutrition, intolerance of oral anticancer agents, dehydration, and hospitalization [32].

The negative impact of tumor organoid factors on the contractile SMC phenotype was further supported by an increase in the SMC proliferation rate and the upregulation of S100A4. Our current understanding of non-vascular SMC proliferation under pathological conditions predominantly originates from studies investigating the role of inflammation in airway and intestinal SMCs. Both chronic airway diseases (e.g., asthma and chronic obstructive pulmonary disease) and inflammatory bowel diseases (e.g., Crohn’s disease) are characterized by the presence of inflammation, which promotes SMC proliferation [33,34,35,36,37,38]. In particular, the inflammatory cytokines tumor necrosis factor alpha (TNF-α) and IL-1β have been shown to induce airway and intestinal SMC proliferation both in vitro and in vivo [35,36,37,39]. This may indicate that visceral SMCs, regardless of the location (i.e., wall of the urinary bladder, uterus, stomach, intestine, or airway), respond similarly to these pro-inflammatory cytokines that affect the SMC phenotype and function. In line with this, we previously showed that pancreatic tumor organoids from cachectic and non-cachectic pancreatic cancer patients expressed variable levels of known cachexia-associated cytokines, including IL-6, TNF-α, IL-8, IL-1α, IL-1β, Mcp-1, and LIF [18]. Furthermore, increasing evidence indicates that the enhanced catabolism experienced by cachectic cancer patients is mediated primarily by increases in pro-inflammatory cytokines, including TNF-α, IL-1, and IL-6 [40]. This further supports the notion that pro-inflammatory cytokines may be responsible for the observed loss of the contractile phenotype and induction of SMC proliferation. Future studies should focus on the identification of these tumor-secreted cytokines and the potential causal effects of these identified factors on visceral SMCs.

Beyond the contractile and proliferative phenotype, SMCs can also participate in and coordinate the inflammatory response by the synthesis and secretion of several signaling molecules, such as pro-inflammatory cytokines, chemotaxis-associated molecules, and growth factors. In the present work, we showed that tumor organoid factors from non-cachectic patients significantly induced IL-8 expression and tended to increase IL-6 expression. Although unexpected, these data are in line with previous studies that revealed the IL-1β-induced upregulation and release of several pro-inflammatory cytokines, including IL-6, IL-8, and Mcp-1, in visceral SMCs [41,42,43]. We previously showed that tumor organoids from non-cachectic pancreatic cancer patients expressed higher IL-1β mRNA levels compared to tumor organoids from cachectic patients [18]. These data suggest that tumor-secreted cytokines may directly induce intracellular changes in SMCs, resulting in the synthesis and secretion of pro-inflammatory cytokines. These alterations can contribute to a persistent systemic inflammatory condition such as cancer cachexia.

Whereas the walls of many visceral organs are surrounded by well-developed smooth muscle layers that enable endured constriction or dilation, the role and behavior of visceral smooth muscle under pathological conditions remain poorly investigated. This is particularly due to difficulties in obtaining human visceral smooth muscle tissue of the organ of interest and the establishment of primary visceral SMC lines that retain a contractile phenotype in culture. In vitro, SMCs are known to modulate uncontrollably towards a synthetic phenotype under standard culture conditions that lack the physiological microenvironment that normally keeps the cells in a contractile phenotype [44]. Moreover, the phenotype and function of SMCs are highly sensitive to environmental stimuli. To surpass these limitations, we used our recently developed human visceral SMC culture model in which SMCs can be kept in either a highly contractile state or modulated towards the synthetic phenotype in a controlled way [19]. This model greatly contributed to our current observations and the relevance of our findings with respect to the human in vivo situation. Nevertheless, contractile functionality cannot be assessed in this in vitro model and data regarding the functionality of the GI tract are lacking in our validation study. This should be integrated in future studies to be able to further explore the link between cachexia and smooth muscle dysfunction and the possible consequences, including malnutrition.

## 5. Conclusions

In conclusion, our data reveal that tumor-derived factors from cachectic patients induce the phenotypic modulation of SMCs towards the synthetic phenotype. Consistent with this, we observed the increased proliferation of the intestinal SMCs of patients with a low skeletal muscle index. Since the loss of the SMC contractile phenotype and increased proliferation are known to impair the contractile functionality of the intestinal smooth musculature, this tumor-induced effect on SMCs may contribute to the frequently reported GI symptoms in cachectic cancer patients.

## Figures and Tables

**Table 1:** Patient characteristics.

	Total (*n* = 22)	High L3-SMI (*n* = 11)	Low L3-SMI (*n* = 11)	*p*-Value
Male (*n*,%)	10 (45.5%)	5 (45.5%)	5 (45.5%)	*p* = 1.00
Age (years)	67.8 (±8.5)	67.8 (±7.2)	67.8 (±10.0)	*p* = 1.00
BMI (kg/m^2^)	23.9 (±2.9)	25.3 (±3.0)	22.5 (±1.9)	*p* = 0.015
Weight loss * (%)	9.4 (±7.4)	8.2 (±7.0)	10.7 (±7.9)	*p* = 0.44
MUST score ≥ 2^#^ (*n*,%)	8 (36.4%)	3 (30.0%)	5 (45.5%)	*p* = 0.66
L3 skeletal muscle index (cm^2^/m^2^)				
Male	49.3 (±4.3)	52.4 (±3.6)	46.1 (±1.9)	*p* = 0.0084
Female	41.9 (±8.0)	48.1 (±5.5)	35.7 (±4.1)	*p* = 0.0013

* Weight loss over the past 6 months; # missing data, *n* = 1.

## Data Availability

Data, analytic methods, and study materials will be made available to other researchers.

## Supplementary Materials

The following supporting information can be downloaded at: https://www.mdpi.com/article/10.3390/cancers16030542/s1, File S1: Raw Data WB analysis; Table S1: qRT-PCR primers; Table S2: L3-SMI in patients with pancreatic cancer; Video S1: Human visceral SMC proliferation—control. Human visceral SMCs were cultured for 3 days on BME-coated surfaces in control medium containing 2% (*v*/*v*) FBS and 50% (*v*/*v*) DMEM/F12. Scale bar = 400 μm; Video S2: Human visceral SMC proliferation—PANCO-9a. Human visceral SMCs were cultured for 3 days on BME-coated surfaces in medium containing 2% (*v*/*v*) FBS and 50% (*v*/*v*) PANCO-9a-derived CM. Scale bar = 400 μm; Video S3: Human visceral SMC proliferation—PANCO-11a. Human visceral SMCs were cultured for 3 days on BME-coated surfaces in medium containing 2% (*v*/*v*) FBS and 50% (*v*/*v*) PANCO-11a-derived CM. Scale bar = 400 μm; Video S4: Human visceral SMC proliferation—PANCO-12a. Human visceral SMCs were cultured for 3 days on BME-coated surfaces in medium containing 2% (*v*/*v*) FBS and 50% (*v*/*v*) PANCO-12a-derived CM. Scale bar = 400 μm; Video S5: Human visceral SMC proliferation—PANCO-17a. Human visceral SMCs were cultured for 3 days on BME-coated surfaces in medium containing 2% (*v*/*v*) FBS and 50% (*v*/*v*) PANCO-17a-derived CM. Scale bar = 400 μm.
