# Supplementary material for: Pancreatic Tumor Organoid-Derived Factors from Cachectic Patients Disrupt Contractile Smooth Muscle Cells
Source: Cancers (Basel). 2024 Jan 26;16(3):542. doi: 10.3390/cancers16030542 (PMC10854749; doi:10.3390/cancers16030542)
Supplement: Supplementary file 1 [file cancers-16-00542-s001.zip › Supplementary Tables.pdf]

Supplemental table

Table S1: qRT-PCR primers

Gene	Full name	Primer Sequence
ACTG2	γ-smooth muscle actin	FW: 5′ – CGCCCTCGCCACCAG – 3′
		RV: 5′ – CCTTGGGATTTAGGGGAGCC – 3′
ATROGIN	Atrogin-1/Muscle atrophy F-box protein	FW: 5′ – AGTTTCGTGAGCGACCTCAG – 3′
		RV: 5′ – TGGTGGAAATACTGAGTTTTGGT – 3′
COL1A1	Collagen Type I Alpha 1 Chain	FW: 5′ – GGTCAGATGGGCCCCCG – 3′
		RV: 5′ – GCACCATCATTTCCACGAGC – 3′
COL3A1	Collagen Type III Alpha 1 Chain	FW: 5′ – GAAAGATGGCCCAAGGGGTC – 3′
		RV: 5′ – TATACCTGGAAGTCCGGGGG – 3′
ELN	Elastin	FW: 5′ – TGTCTGCAGCCCCTTCTGTG – 3′
		RV: 5′ – GGCACTTTCCCAGGCTTCA – 3′
IL1B	Interleukin 1 beta	FW: 5′ – CTGAGCTCGCCAGTGAAATG – 3′
		RV: 5′ – TTTAGGGCCATCAGCTTCAAA – 3′
IL6	Interleukin 6	FW: 5′- TACCCCCAGGAGAAGATTCC – 3′
		RV: 5′ – TTTCAGCCATCTTTGGAAGG – 3′
IL8	Interleukin 8	FW: 5′ – CTGGCCGTGGCTCTCTTG – 3′
		RV: 5′ – TTAGCACTCCTTGGCAAAACTG – 3′
MCP1	Monocyte chemotactic protein 1	FW: 5′ – TCACCTGCTGTTATAACTTCAC – 3′
		RV: 5′ – CAATGGTCTTGAAGATCACAG – 3′
S100A4	S100 Calcium Binding Protein A4	FW: 5′ – TCTTGGTTTGATCCTGACTGCT – 3′
		RV: 5′ – GCCCGAGTACTTGTGGAAGG – 3′
B2M	Beta-2-microglobulin	FW: 5′ – TCCATCCGACATTGAAGTTG – 3′
		RV: 5′ – CGGCAGGCATACTCATCTT – 3′
CYPA	Cyclophylin A	FW: 5′ – CTCGAATAAGTTTGACTTGTGTTT – 3′
		RV: 5′ – CTAGGCATGGGAGGGAACA – 3′

Table S2: L3-SMI in patients with pancreatic cancer

Patient ID	Gender	L3-SMI	Median cut-off	Sarcopenia/cachectic
43	Female	57,460938	>42,4973	High L3-SMI
48	Female	42,752629	>42,4973	High L3-SMI
49	Female	43,848954	>42,4973	High L3-SMI
74	Female	34,90625	<42,4973	Low L3-SMI
75	Male	49,005682	>48,7453	High L3-SMI
80	Male	44,969136	<48,7453	Low L3-SMI
84	Female	42,241946	<42,4973	Low L3-SMI
91	Male	47,859691	<48,7453	Low L3-SMI
108	Female	35,831038	<42,4973	Low L3-SMI
111	Female	45,343698	>42,4973	High L3-SMI
113	Male	50,494192	>48,7453	High L3-SMI
117	Female	30,926119	<42,4973	Low L3-SMI
118	Female	38,083498	<42,4973	Low L3-SMI
120	Male	57,252872	>48,7453	High L3-SMI
146	Male	45,440877	<48,7453	Low L3-SMI
148	Female	32,170067	<42,4973	Low L3-SMI
169	Female	51,52376	>42,4973	High L3-SMI
181	Male	55,154321	>48,7453	High L3-SMI
182	Male	48,484848	<48,7453	Low L3-SMI
183	Male	43,974907	<48,7453	Low L3-SMI
188	Female	47,876239	>42,4973	High L3-SMI
192	Male	50,305568	>48,7453	High L3-SMI
